# Effects of Dietary Cottonseed Protein Concentrate Levels on Growth Performance, Health Status, Flesh Quality and Intestinal Microbiota of Grass Carp (*Ctenopharyngodon idellus*)

**DOI:** 10.3390/metabo12111046

**Published:** 2022-10-30

**Authors:** Guoqing Liu, Meng Zhou, Xiaoyu Wang, Xiangjie Mao, Xianmei Long, Shouqi Xie, Dong Han, Qingsong Tan

**Affiliations:** 1 Engineering Research Center of Green Development for Conventional Aquatic Biological Industry in the Yangtze River Economic Belt, Ministry of Education/Key Laboratory of Freshwater Animal Breeding, Ministry of Agriculture/Hubei Provincial Engineering Laboratory for Pond Aquaculture, College of Fisheries, Huazhong Agricultural University, Wuhan 430070, China; 2 State Key Laboratory of Freshwater Ecology and Biotechnology, Institute of Hydrobiology, Chinese Academy of Sciences, Wuhan 430072, China

**Keywords:** cottonseed protein concentrate, protein requirement, muscle texture, antioxidant capacity, metabolic function

## Abstract

The aim of this study was to evaluate the nutritional value of cottonseed protein concentrate (CPC) as a single dietary protein source and the optimal protein level for grass carp (*Ctenopharyngodon idellus*). An 8-week feeding trial was conducted by feeding juvenile grass carp (initial body weight: 4.68 ± 0.01 g) with six experimental diets containing graded levels of protein provided by CPC. The results showed that the optimal CPC level (CPC4) improved the growth performance and health status of grass carp. The optimal dietary protein level was estimated to be 38.61 and 38.66% based on specific growth rate (SGR) and feed efficiency (FE), respectively. The CPC4 group significantly increased the total antioxidant capacity (T-AOC) content and glutathione peroxidase (GSH-Px) activity in the hepatopancreas (*p* < 0.05). In addition, the CPC4 group increased the muscle T-AOC and glutathione (GSH) content and improved muscle hardness, and the gene expression of MRFs, *fgf6a*, *myhc-7*, *myhc-1*, *myhc-4*, *igf-II*, and *tor* was upregulated while *mstn* gene expression was downregulated (*p* < 0.05). Correlation analysis revealed that the optimal dietary CPC level promoted grass carp growth, health, and flesh quality by regulating the relative abundance of intestinal microbes. Furthermore, CPC6 upregulated the ko00480 (Glutathione metabolism) and ko00620 (Pyruvate metabolism) pathways compared to CPC1 (*p* < 0.05), possibly indicating that low dietary CPC levels adversely affected amino acid metabolism in the intestinal microbiota of grass carp, while a high level of CPC will meet the metabolic needs of the body by increasing the utilization of energy.

## 1. Introduction

Protein is the most expensive ingredient in aquafeed and profoundly impacts the growth and health of aquatic animals. However, the shortage of traditional protein sources, such as fishmeal and soybean meal, hinders the development of the global feed industry [1]. Cottonseed protein plays an essential role in aquaculture as a plant protein source [2]. However, cottonseed contains gossypol and other anti-nutritional factors, limiting its inclusion level in a diet [2]. Through a series of processing, including dehairing, dehulling, low-temperature oil extraction, and solvent extraction, the cottonseed is processed into cottonseed protein concentrate (CPC), which significantly reduce the anti-nutritional factors [3]. Currently, researchers are extensively investigating the potential of CPC as alternative materials in diets [3,4,5], aiming to alleviate protein shortages. CPC can replace 40–45% of fishmeal in largemouth bass (*Micropterus salmoides*) diets without affecting largemouth bass growth performance [3,4]. Likewise, CPC can replace 24% of the fishmeal in pearl gentian grouper (*Epinephelus fuscoguttatus*♀ × *Epinephelus lanceolatu*♂) and promote growth performance [5]. However, nutritional trials using CPC as the sole protein source are uncommon, which is desirable to evaluate the biological value of CPC.

Grass carp (*Ctenopharyngodon idellus*) is herbivorous and feeds on certain water plants in its natural environment [6]. The grass carp culture shares a long history and the largest freshwater aquaculture production in China due to the euryphagic feeding habit and delicious meat of grass carp, which is one of the “four major Chinese carps”. Grass carp has been introduced into more than 100 countries around the world for food, weed control, or research [7]. According to FAO statistics, the global aquaculture production in 2020 was 87.5 million tons, among which grass carp production was 5.79 million tons (the top three countries in production are China, Bangladesh, and Iran), accounting for about 6.62% [8]. Nowadays, grass carp culture relies mainly on feeding with compound feed to meet the market demand. Previous studies have determined protein requirements for grass carp based primarily on semi-purified feeds using fishmeal or casein, which indicated that the optimal protein level vary according to the protein materials and fish growth stages [9,10,11,12]. Moreover, precise nutrition for aquatic animals requires accurate knowledge regarding the protein sources and their optimal inclusion levels, which has progressively attracted attention from scholars. Therefore, evaluating the optimal dietary protein level in grass carp diets based on CPC as a protein source must be emphasized under the circumstance of precision nutrition.

Flesh quality has always been the most valued part by consumers. The indicators for evaluating flesh quality include cooking loss, texture properties (including hardness, cohesiveness, gumminess, springiness, resilience, and chewiness), pH, and antioxidant capacity [13]. Muscle growth is a dynamic process, including muscle hyperplasia and hypertrophy [14], which is affected by multiple regulatory factors such as muscle regulatory factors (MRFs), insulin-like growth factors (IGFs), fibroblast growth factor 6 (*fgf6*), myostatin (*mstn*), and target of rapamycin (*tor*) [15,16]. There have been many achievements in regulating flesh quality by dietary nutrients in mammals, but the regulation of flesh quality in aquatic animals still needs to be investigated in depth. Existing studies have shown that amino acid supplementation at an appropriate level can improve the flesh quality of grass carp [13] and hybrid bagrid catfish (*Pelteobagrus vachelli*♀ × *Leiocassis longirostris*♂) [15]. Grass carp that ate fava beans gained better flesh quality, mainly due to increased muscle hardness [17,18]. Furthermore, a previous study showed that replacing rapeseed meal and cottonseed meal with DDGS induced the myosin isoforms transformation of grass carp, altering the muscle texture properties, histological muscle properties, and gene expression of MRFs [16,19]. A recent study showed that feeding grass carp (6.80 ± 0.10 g) with graded protein level (soybean meal) diets improved flesh quality at the appropriate protein level [20]. Likewise, in a study of large grass carp (264.11 ± 0.76 g), optimal protein levels (fish meal, casein and gelatin) also improved muscle texture and antioxidant capacity [12]. Therefore, the effect of varying dietary protein levels from CPC on the muscle histology, myosin heavy chain (*myhc*) gene expression levels, and muscle texture properties deserves investigation, for a more comprehensive nutritional value evaluation of CPC.

The homeostasis of intestinal microbes regulated by nutrients has crucial guiding significance for human health [21]. Rapid, low-cost, and precise DNA sequencing methods, such as those offered by Illumina (San Diego, CA, USA), are becoming increasingly popular and extensively used to examine intestinal microbial composition [21]. Recently, the importance of intestinal microbes in aquatic animals has been paid more attention to by researchers [4,5]. However, previous studies focused on the diversity in intestinal microbial composition after fishmeal replacement or exposure to specific factors [3,22]. Research on the effect of protein level on fish intestinal microbiota has only been reported for Songpu mirror carp (*Cyprinus carpio*) [23]. In addition, the mammalian gut–muscle axis also have received attention [24], but these studies are still in their infancy in aquatic animals. In this regard, a crosstalk analysis between the homeostasis of intestinal microbes and the growth and health status will beneficial, to illustrate the regulative mechanism of the CPC protein level.

To sum up, given the importance of protein resources, the urgent need of farmers for the rapid and healthy growth of fish, and the pursuit of superior flesh quality by consumers, it is necessary to evaluate the nutritional value of CPC as a single protein source and to quantify the optimal protein level for grass carp. Using CPC as a protein source, six treatments were designed containing a gradient of protein levels, subsequently conducting an 8-week feeding trial to investigate the effects of these protein levels on the growth, health, flesh quality, and intestinal microbiota of grass carp.

## 2. Materials and Methods

### 2.1. Experimental Diets

Dietary treatments, ingredients, and chemical composition are shown in Table 1. CPC was used as a protein source, starch was used as a carbohydrate source, and soybean oil and fish oil were used as lipid sources to prepare the six experimental diets with different protein contents. The protein contents of the six experimental diets, named CPC1, CPC2, CPC3, CPC4, CPC5, and CPC6, were 24.80%, 30.51%, 33.68%, 37.69%, 41.43%, and 45.61% (expressed in dry matter), respectively; also, 0.1% yttrium oxide (Y_2_ O_3_) was added to the CPC experimental diets for the determination of apparent digestibility coefficients (ADCs) [16]. The CPC was provided by Xinjiang Jinlan Plant Protein Co., Ltd., Shihezi, Xinjiang, China, which contained 61.51% crude protein, 2.36% crude lipid, 5.35% moisture, 5.70% ash, 4.82% crude fiber, 19.55 MJ/kg gross energy, 0.05% tannin, 1.29 mg/kg sterculic acid, and 285 mg/kg free gossypol. According to the instructions provided by the manufacturer, the CPC is made as follows: Briefly, the regular glanded cottonseeds were first crushed and sieved to remove the hulls; then, the kernels were softened under a certain moisture content and rolled into 0.4 mm-thick flakes and dried. The softening, rolling, and drying were processed at a low temperature (60–70 °C). The dried flakes were extracted with mixed solvents of methanol and *n*-hexane in different concentrations for oil extraction and dephenolization, and then subjected to rapid low-temperature drying (not higher than 90 °C) to obtain the final product. The raw materials in the diets were ground and screened through an 80-mesh sieve, then accurately weighed, and finally mixed uniformly using a M-256 mixer (South China University of Technology, Guangzhou. Pellets (diameter: 2.0 mm) were extruded using a laboratory feed extruder (feed moisture: 21%, expansion temperature: 130 °C), air dried, and then stored at −20 °C.

### 2.2. Fish and Feeding Trial

Juvenile grass carp were purchased from Xinzhou Fisheries Co. Ltd. (Wuhan, China). Prior to the growth trial, the fish were acclimated to the culture conditions and were fed with an even mixture of the six diets. After 2 weeks of acclimation, 540 fish were divided into 18 tanks in a flow-through culture system at random, with 30 fish (initial body weight, 4.68 ± 0.01 g) in each tank (water volume: 300 L, water exchange: 1 L/min). Each diet was delivered randomly to three tanks. The growth trial lasted for 8 weeks. During the growth trial, fish were hand-fed twice a day (08:30 and 15:30). The feed consumed in each tank was recorded daily. Uneaten feed was collected 1 h after feeding and dried in an oven at 60 °C to calibrate grass carp food intake. Throughout the experiment, an air pump was used to ensure that the dissolved oxygen in the water tank was above 6 mg/L; furthermore, the water temperature was maintained at 27 to 28 °C, pH was 7.3 to 7.6, and the natural photoperiod was applied.

### 2.3. Sample Collection

At the conclusion of the 8-week growth trial, fish were deprived of food for 24 h. All fish in each tank were counted and collectively weighed. Five fish, randomly selected from each tank, and anesthetized with diluted MS222 (75 mg/L, Aladdin, Shanghai, China), were weighed and their body length measured individually. Then, blood samples were collected from the caudal vein, precipitated at room temperature for 30 min, and serum samples were separated by centrifugation at 4 °C for 10 min at 3000× *g* and stored at −80 °C for analysis. Afterward, the viscera were removed and weighed; then, the hepatopancreas and mesenteric fat were further dissected and weighed. Tissue samples (hepatopancreas and white muscle under the dorsal fin) were collected, fast-frozen in liquid nitrogen, and stored at −80 °C for RNA extraction and enzyme activity evaluation. The intestinal contents of the CPC1, CPC4, and CPC6 groups of fish were collected (in order to explore the effects of low dietary protein level, optimal dietary protein level, and high dietary protein level on the intestinal flora of grass carp), with three biological replicates in each group, and the intestinal contents of six grass carp were collected and mixed as a sample for each biological replicate. The samples were fast-frozen in liquid nitrogen and stored at −80 °C for analysis of intestinal microbial diversity. Furthermore, the hepatopancreas and muscle were sampled and fixed in 4% paraformaldehyde and stored in 70% ethanol for histological observations. Three fish were randomly selected from each tank as samples for muscle texture analysis. In the last 4 weeks of the growth trial, feces were siphoned from each tank twice daily at 2 h after feeding, oven-dried at 60 °C, and then stored at −20 °C for subsequent nutrient apparent digestibility determination. Furthermore, five fish were randomly selected from each tank and stored at −20 °C for proximate analysis of the body. All operating procedures were carried out on ice.

### 2.4. Sample Analysis 

#### 2.4.1. Determination of Serum Biochemical Indices

Serum total protein (TP), glucose (GLU), urea nitrogen (UN), high-density lipoprotein (HDL), low-density lipoprotein (LDL), triglycerides (TG), and cholesterol (CHOL) were detected with an automated biochemical analyzer (Abbott Aeroset^®^, Abbott Laboratories, Chicago, IL, USA) using commercial test kits. Serum activities of lysozyme (LYS), complement 3 (C3), and the content of immunoglobulin M (IgM) were determined by commercially available kits (the product numbers are ml036413, ml092636, and ml092683, respectively) purchased from Shanghai Enzyme-linked Biotechnology Co. Ltd. (Shanghai, China), according to the manufacturer’s instructions.

#### 2.4.2. Proximate Composition

Proximate analysis was conducted for the experimental feed, feces, dorsal muscle, and whole fish according to the standard procedures of AOAC [25]. Briefly, samples were dried to a constant weight at 105 °C to estimate the moisture content. Crude protein was measured using the Kjeldahl method by determining nitrogen (*n* × 6.25) after acid digestion. Crude lipid was determined by ether extraction using the Soxhlet method. Ash was measured in a muffle furnace at 550 °C for 5 h. 

#### 2.4.3. Enzyme Activity of Muscle and Hepatopancreas

The enzyme activities of alanine aminotransferase (ALT), aspartate aminotransferase (AST), superoxide dismutase (SOD), catalase (CAT), total antioxidant capacity (T-AOC), and glutathione peroxidase (GSH-Px), as well as the reduced glutathione (GSH) and malondialdehyde (MDA) contents in the muscle and hepatopancreas were determined using commercial kits (Jian Cheng Bioengineering Institute, Nanjing, China).

#### 2.4.4. Apparent Digestibility

According to a previous study [16], the experimental diets and feces were digested with nitric acid, and the Yttrium content was detected using an IRIS Advantage inductively coupled plasma (ICP) atomic emission spectrophotometer (Thermo Jarrell Ash Corporation, Boston, MA, USA) for the calculation of the apparent digestibility coefficients. 

#### 2.4.5. Histological Observation

Paraformaldehyde-fixed muscle and hepatopancreas tissues were dehydrated with gradient grades of ethanol, followed by embedding with paraffin. Thick sections (7 μm) were prepared and stained with hematoxylin–eosin (HE). The stained samples were observed under a light microscope. The number of muscle fibers and diameter were measured using an M-Shot image analysis system (Micro-Shot, Guangzhou, China). 

#### 2.4.6. Muscle Textural Properties

Cooking loss of muscle was determined based on a previous study [16]. In short, 2–3 g of muscle was weighed, wrapped in gauze, and then cooked in boiling water (100 °C) for 5 min; then, the muscle was taken out and weighed after removing the surface water with an absorbent paper. Cooking loss was reported in terms of weight loss during heat processing and expressed as % of the initial sample weight. Texture profile analysis (TPA) of the muscle was determined using a TA.XT Plus texture analyzer (Stable Micro Systems, Godalming, UK) equipped with a flat-bottomed cylindrical probe *p*/36 R (20 mm diameter), as previously reported by Kong et al. [19]. The measurements were performed on three fish from each tank.

#### 2.4.7. Gene Expression Quantification

Total RNA was extracted using Trizol™ reagent (Takara, Dalian, China). The RNA integrity and quantity were assessed by Evolution-Capt image analysis software after 1% agarose gel electrophoresis and a 260/280 nm absorbance ratio (NanoDrop^®^ ND-1000, Thermo Fisher Scientific, Waltham, MA, USA). High-quality RNA with a 28 S/18 S ratio ≥2 and a 260/280 ratio of 1.9–2.1 was used for further quantification. Subsequently, cDNA was synthesized using a PrimeScript RT reagent kit with a gDNA eraser (Yeasen, Shanghai, China). 

Primers for quantitative real-time PCR (qRT-PCR) were designed according to the sequences existing in National Center for Biotechnology Information Search database (Table 2). The qRT-PCR reaction was performed using a Hieff^®^ qPCR SYBR^®^ Green Master Mix (No Rox) (Yeasen, Shanghai, China) on a quantitative thermal cycler (Light Cycler 480 II, Roche). The qRT-PCR reaction procedures were as follows: pre-incubation at 95 °C for 5 min, followed by 40 cycles of 95 °C for 10 s, and annealing temperature (corresponding specific primer pairs) for 20 s and 72 °C for 20 s. Melting curve analysis was performed to ensure that only one fragment was amplified. The relative expression levels of the target genes were calculated by normalization via the 2^−ΔΔCT^ method, as described by Pfaffl [26], and using *β-actin* and *ef1α* as the reference genes.

#### 2.4.8. Intestine Microbiome Analysis 

Microbial community DNA was extracted from intestinal contents using the E.Z.N.A.^®^ Soil DNA Kit (Omega Bio-Tek, Norcross, GA, USA). The integrity of the DNA was checked on 1% agarose gel, while the DNA concentration and purity were detected on a NanoDrop 2000 UV–vis spectrophotometer (Thermo Scientific, Wilmington, NC, USA). After amplification of the hypervariable V3–V4 regions of the bacterial 16 S rRNA gene on an ABI GeneAmp^®^ 9700 PCR thermocycler (ABI, Foster, CA, USA) using primer pairs 338 F (5′-ACTCCTACGGGAGGCAGCAG-3′) and 806 R (5′-GGACTACHVGGGTWTCTAAT-3′), the PCR products were sequenced by the Illumina MiSeq PE300 platform (Illumina, San Diego, CA, USA). The raw reads from this study were deposited in the NCBI Sequence Read Archive (SRA) database (Accession Number: PRJNA826686).

The raw reads of 16 S rRNA gene sequencing were demultiplexed, quality filtered, and merged by fastp (version 0.20.0, https://github.com/OpenGene/fastp) and FLASH (version 1.2.7, https://ccb.jhu.edu/software/FLASH/index.shtml). Bioinformatic statistical analysis of the operational taxonomic units (OTUs) at 97% similarity level was performed using Uparse (version 7.0.1090, http://www.drive5.com/uparse/). Compared with the 16 S rRNA database (Silva 138), the RDP classifier Bayesian algorithm was used to perform taxonomic analysis on the representative sequences of OTUs (confidence threshold was 0.7). The Alpha diversity index under different random sampling was calculated using mothur (version 1.30.2, https://www.mothur.org/wiki/Download_mothur) and the difference between groups was compared by Welch’s *t*-test. Statistical analysis and graphing of the PCoA (principal co-ordinates analysis) was performed using the R language (version 3.3.1, https://www.r-project.org/); ANOSIM analysis using the weighted UniFrac metric was also performed to judge whether the groupings are meaningful. One-way ANOVA was used to discriminate between-group differences in Firmicutes, Bacteroidetes, and F/B values. Spearman coefficients were used to indicate the correlation between clinical factors and species. In addition, LEfSe (linear discriminant analysis of effect size) performs linear discriminant analysis (LDA) according to the degree of influence of species composition on samples, identifying those species with significant differences. Finally, the 16 S taxonomic lineage based on the Silva database was transformed into the taxonomic lineage of prokaryotes in the KEGG database by Tax4 Fun. KEGG functional annotation was performed on the 16 S RNA gene sequence.

### 2.5. Statistical Analysis

Data were expressed as the mean ± standard error (SE). The data analysis was carried out with the SPSS computer program, version 26 (IBM, Armonk, NY, USA). After confirmation of the normality and homogeneity of variance (Levene’s test) of the results, the effect of diet treatments was identified by one-way analysis of variance (ANOVA), and the difference between groups was further compared by Duncan’s test. The difference was considered significant at *p* < 0.05. The data were compared with orthogonal polynomials, and if significance (linear, quadratic, or cubic) was detected, the model was further fitted by regression analysis. A two-slope broken-line linear (2 SBL-LL) model was also tested if the quadratic or cubic regression was significant. The R square (R^2^) was used for optimal regression selection to detect the optimal protein level for dependent variables. The dietary protein requirement (provided by CPC) of grass carp was obtained by the broken-line analysis based on SGR and FE.

## 3. Results

### 3.1. Growth Performance, Apparent Digestibility, and Morphology Parameters

**Figure 1:**
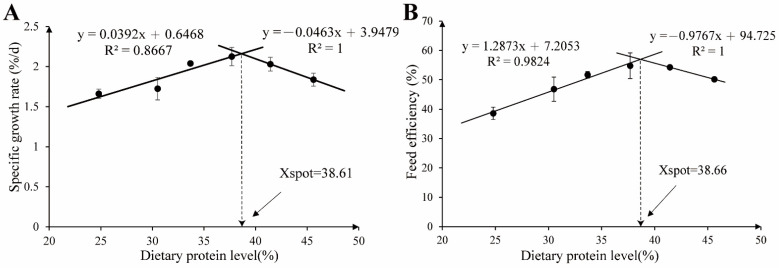
Broken-line analysis of the relationship between the dietary protein level and growth performance of grass carp. (**A**) The regression analysis between the specific growth rate and dietary protein level. (**B**) The regression analysis between the feed efficiency and dietary protein level.

The effects of graded dietary protein levels on growth performance, apparent digestibility, and morphological parameters are presented in Table 3. After 8 weeks of feeding, the final body weight (FBW) of the grass carp was about three times the initial body weight, and there was no significant difference in survival rate among treatments (*p* > 0.05). As the dietary protein content increased gradually, the FBW, specific growth rate (SGR), and feed efficiency (FE) all exhibited an increasing first and then a decreasing trend in the quadratic model (*p* < 0.05, R^2^ = 0.541, 0.545, and 0.642), all of which showed the highest values in the CPC4 group. The feeding rate (FR) and protein efficiency ratio (PER) showed a linear downward trend with increasing protein levels (*p* < 0.05, R^2^ = 0.410 and 0.528). Furthermore, as dietary protein levels increased, the apparent digestibility coefficient of the dry matter (ADC_d_) and morphological parameters (including condition factor (CF), hepatosomatic index (HSI), and mesenteric fat index (MFI)) first increased and then decreased in the quadratic model (*p* < 0.05, R^2^ = 0.881, 0.603, 0.777, and 0.741), while the apparent digestibility coefficient of the protein (ADC_p_) exhibited a rising linear model (*p* < 0.05, R^2^ = 0.734). Based on the broken-line analysis of SGR and FE, the optimal dietary protein (CPC) level of the grass carp was estimated to be 38.61 and 38.66%, respectively (Figure 1).

### 3.2. Body and Dorsal Muscle Proximate Composition

As shown in Table 4, the crude lipid content in the whole body increased linearly (R^2^ = 0.375), while the muscle protein content increased first and then decreased in the quadratic model in response to the increasing protein level (*p* < 0.05, R^2^ = 0.331). No other significant difference in the proximate composition of the whole body and dorsal muscle was observed between treatments (*p* > 0.05).

### 3.3. Serum Biochemical Indices and Immune Enzyme Activity

The biochemical indices and immune parameters in the serum of grass carp are presented in Table 5. The serum TP showed a linearly increasing trend as the dietary protein level increased (R^2^ = 0.291) but was not significantly different among all treatments. The contents of serum UN and IgM also increased linearly with dietary protein levels (*p* < 0.05, R^2^= 0.696 and 0.650). In contrast, the contents of TG, LDL, and C3 in the serum increased first and then decreased, responding to the increasing dietary protein level in the quadratic model (*p* < 0.05, R^2^ = 0.710, 0.300, and 0.471, respectively). However, the dietary protein level did not affect the contents of serum GLU, CHOL, HDL, and LYS of grass carp (*p* > 0.05). 

### 3.4. Antioxidative Capacity, Metabolic Enzymes, and Histological Observation of Hepatopancreas

**Figure 2:**
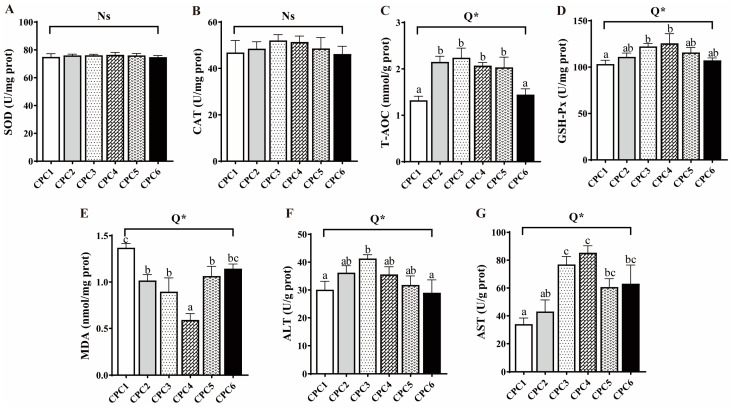
Effects of the dietary protein levels on antioxidative capacity and transaminase activities of grass carp hepatopancreas. (**A**–**G**) SOD: superoxide dismutase; CAT: catalase; T-AOC: total antioxidant capacity; GSH-Px: glutathione peroxidase; MDA: malondialdehyde; ALT: alanine aminotransferase; AST: aspartate aminotransferase. All data were the means of three parallel tanks (*n* = 3). Mean values not sharing a common superscript in the same row are significantly different (*p* < 0.05), while mean values in the same row without any superscript are not different. If statistical significance (*p* < 0.05) was detected, the model that fits best with the data was selected. Ns = No structure (*p* > 0.05); Q = Quadratic; * *p* < 0.05.

As shown in Figure 2, hepatopancreatic T-AOC, GSH-Px, AST, and ALT activities significantly increased first and then decreased in response to the increasing dietary protein level in the quadratic model (R^2^ = 0.666, 0.461, 0.558, and 0.386, respectively). The activities of T-AOC and ALT peaked in the CPC3 group (*p* < 0.05), while GSH-Px and AST showed the highest values in the CPC4 group (*p* < 0.05). The hepatopancreatic MDA content significantly decreased first and then increased with dietary protein level and was the lowest in the CPC4 group (*p* < 0.05, R^2^ = 0.623). In contrast, SOD and CAT activities were not significantly different among all treatments (*p* > 0.05).

**Figure 3:**
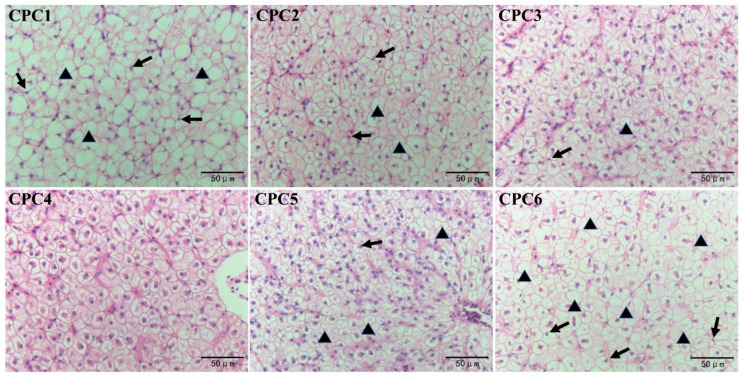
Hepatopancreatic histology of grass carp (hematoxylin and eosin, ×400). Arrows indicate nuclei shifted to the periphery of the hepatocytes; triangles indicate vacuolation.

As shown in Figure 3, the dietary protein level significantly affected the hepatopancreatic microstructure of grass carp. CPC3 and CPC4 groups showed more normal hepatocytes without noticeable swelling and atrophy. However, CPC1 and CPC6 groups showed more abnormal hepatocytes, nuclear migration, cellularity vacuum, and lipid deposition. Some hepatocyte nuclear migration and cell vacuums were observed in the CPC2 and CPC5 groups.

### 3.5. Muscle Texture Analysis

As shown in Table 6, the cooking loss of muscle increased linearly with the dietary protein level (*p* < 0.05, R^2^ = 0.611). The muscle texture parameters, including hardness, cohesiveness, gumminess, and chewiness in the cooked meat, increased first and then decreased in response to the increasing dietary protein level in the quadratic model (*p* < 0.05, R^2^ = 0.687, 0.480, 0.637, and 0.645, respectively). Muscle springiness was not affected by the dietary protein level (*p* > 0.05). Muscle resilience first decreased and then increased in the quadratic model (*p* < 0.05, R^2^ = 0.717), while pH showed a linear downward trend with the dietary protein level (*p* < 0.05, R^2^ = 0.282). 

### 3.6. Antioxidative Capacity and Histological Observation of Muscle

**Figure 4:**
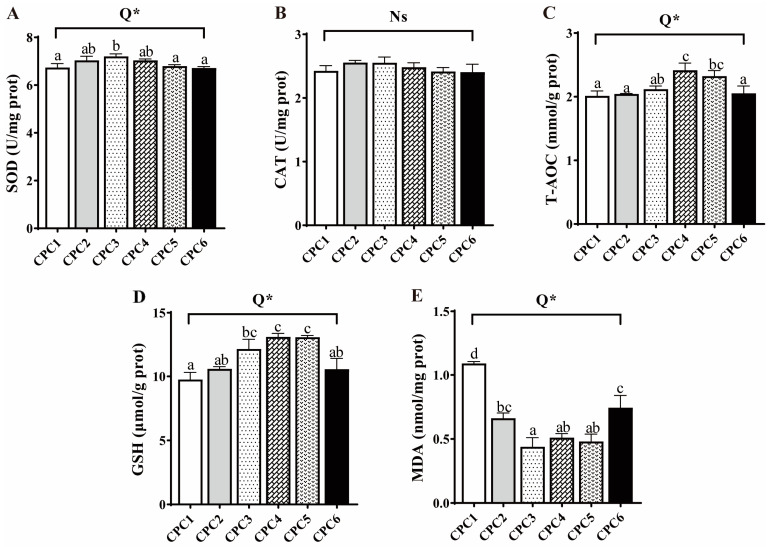
Effects of the dietary protein level on the antioxidant capacity of grass carp muscle. (**A**–**E**) SOD: superoxide dismutase; CAT: catalase; T-AOC: total antioxidant capacity; GSH: glutathione; MDA: malondialdehyde. All data are means of three parallel tanks (*n* = 3). Mean values not sharing a common superscript in the same row are significantly different (*p* < 0.05), while mean values in the same row without any superscript are not different. If statistical significance (*p* < 0.05) was detected, the model that fits best with the data was selected. Ns = No structure (*p* > 0.05); Q = Quadratic; * *p* < 0.05.

The antioxidant parameters, including the activities of SOD, T-AOC, and GSH contents in muscle, significantly increased first and then decreased in response to the increasing dietary protein level in the quadratic model (*p* < 0.05, R^2^ = 0.442, 0.365, and 0.627, respectively), which were the highest in the CPC2 or CPC4 group, respectively (Figure 4). The MDA content decreased first and then increased in the quadratic model (*p* < 0.05, R^2^ = 0.842) responding to the protein level, and was the lowest in CPC3. However, there was no significant difference in muscle CAT activity (*p* > 0.05). 

**Figure 5:**
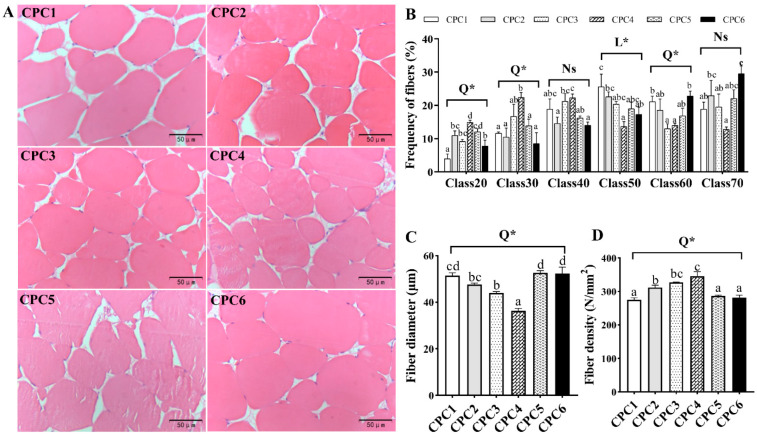
Muscle histology of grass carp fed with different protein levels for eight weeks. (**A**) Observation on muscle fiber of white muscle in grass carp (hematoxylin and eosin, ×400). (**B**) The frequency distribution of the muscle fibers’ diameter classes (d, μm): class 20 = d ≤ 20, class 30 = 20 < d ≤ 30, class 40 = 30 < d ≤ 40, class 50 = 40 < d ≤ 50, class 60 = 50 < d ≤ 60, and class 70 = d > 60. (**C**) The muscle fiber diameter (μm). (**D**) The muscle fiber density (*n*/mm^2^). All data are the means of three parallel tanks (*n* = 3). Mean values not sharing a common superscript in the same row are significantly different (*p* < 0.05), while mean values in the same row without any superscript are not different. If statistical significance (*p* < 0.05) was detected, the model that fits best with the data was selected. Ns = No structure (*p* > 0.05); L = Linear; Q = Quadratic; * *p* < 0.05.

As shown in Figure 5A, dietary protein level affected the microstructure of grass carp dorsal muscle. The frequency distribution of grass carp muscle fibers shows that the CPC4 group was assigned smaller diameters, including class20, class30 (*p* < 0.05, R^2^ = 0.624 and 0.384), and class40 (Figure 5B). Large muscle fibers, including class50, class60 (*p* < 0.05, R^2^ = 0.346 and 0.580), and class70 diameters, were more assigned to other groups (Figure 5B). Further statistical analysis found that fiber diameter (Figure 5C) and muscle fiber density (Figure 5D) exhibited a quadratic model (*p* < 0.05, R^2^ = 0.664 and 0.513), with the largest muscle fiber density and smallest muscle fiber diameter detected in the CPC4 group. 

### 3.7. Muscle-Related Genes Expression

**Figure 6:**
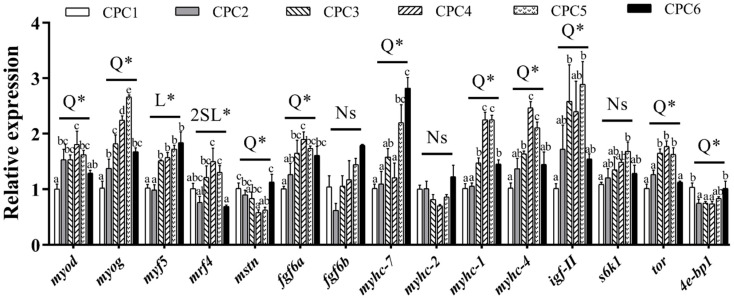
Muscle-related genes expression of grass carp fed with different protein levels for eight weeks. *myod*—myogenic differentiation antigen; *myog*—myogenin; *myf5*—myogenic factor 5; *mrf4*—muscle regulatory factor 4; *fgf6a*—fibroblast growth factor 6 a; *fgf6b*—fibroblast growth factor 6 b; *mstn*—myostatin; *myhc-7*—myosin heavy chain 7; *myhc-2*—myosin heavy chain 2; *myhc-1*—myosin heavy chain 1; *myhc-4*—myosin heavy chain 4; *igf-II*—insulin-like growth factor 2; *tor*—target of rapamycin; *s6k1*—ribosomal protein S6 kinase 1; *4e-bp1*—4 e-binding protein 1; *ef1α*—elongation factor 1-alpha. The cDNAs used to detect *myhc-2* and *myhc-4* were diluted 180 times; others were diluted six times. All data are the means of three parallel tanks (*n* = 3). Mean values not sharing a common superscript in the same row are significantly different (*p* < 0.05), while mean values in the same row without any superscript are not different. If statistical significance (*p* < 0.05) was detected, the model that fits best with the data was selected. Ns = No structure (*p* > 0.05); 2 SL = Two slope broken line-linear ascending and linear descending; L= Linear; Q = Quadratic; * *p* < 0.05.

As shown in Figure 6, the gene expression of myogenic differentiation antigen (*myod*), myogenin (*myog*), *mstn*, *fgf6a*, *myhc-7*, *myhc-1*, *myhc-4*, *igf-II*, *tor*, and 4 e-binding protein 1 (*4e-bp1*) in the muscle all varied in the quadratic models (*p* < 0.05, R^2^ = 0.564, 0.722, 0.443, 0.621, 0.733, 0.631, 0.656, 0.456, 0.785, and 0.567, respectively) as dietary protein level increased. Gene expression of muscle regulatory factor 4 (*mrf4*) varied in the 2 SBL-LL model with increasing dietary protein levels (*p* < 0.05, R^2^ = 0.337), while myogenic factor 5 (*myf5*) showed a linear upward trend (*p* < 0.05, R^2^ = 0.756). The maximum gene expression of *myod*, *mrf4*, *fgf6a*, *myhc-1*, *myhc-4*, and *tor* was detected in the CPC4 group, while gene expression of ribosome S6 protein kinase 1 (s6k1) peaked in the CPC5 group. The minimum gene expression values of *mstn*, *4e-bp1,* and *myhc-2* were detected in the CPC4 group. However, dietary protein levels did not significantly affect the *fgf6b* gene expression levels (*p* > 0.05).

### 3.8. Intestinal Microbiota of Grass Carp

**Figure 7:**
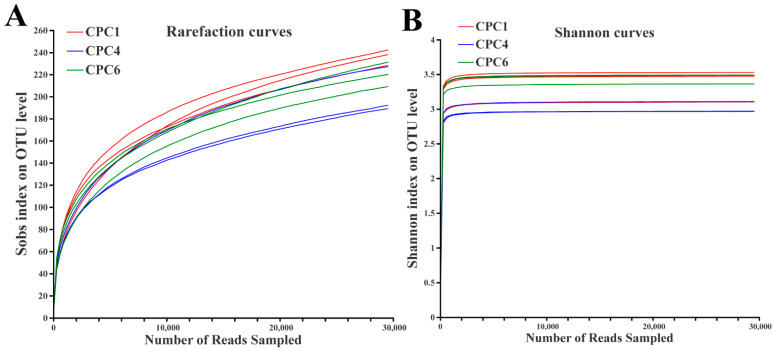
Rarefaction curves (**A**) and Shannon curves (**B**) of the OTUs clustered at a 97% phylotype similarity level of the fish intestinal microbiota from the different dietary protein treatments. The horizontal axis is the number of valid sequences; the vertical axis is the observed number of operational taxonomic units. The Sobs index and Shannon index were used to estimate the richness and diversity of the intestinal microbiota, respectively.

After pyrosequencing, the samples were leveled to 29,566 sequences according to the minimum sample sequence value among the nine samples. All sequences were delineated as OTUs with 97% sequence similarity values, yielding 485 OTUs. Shannon and rarefaction curves tended to approach saturation plateaus, indicating that the microbial diversity and total OTUs in the different samples did not vary with the apparent change in sequencing depth (Figure 7).

**Figure 8:**
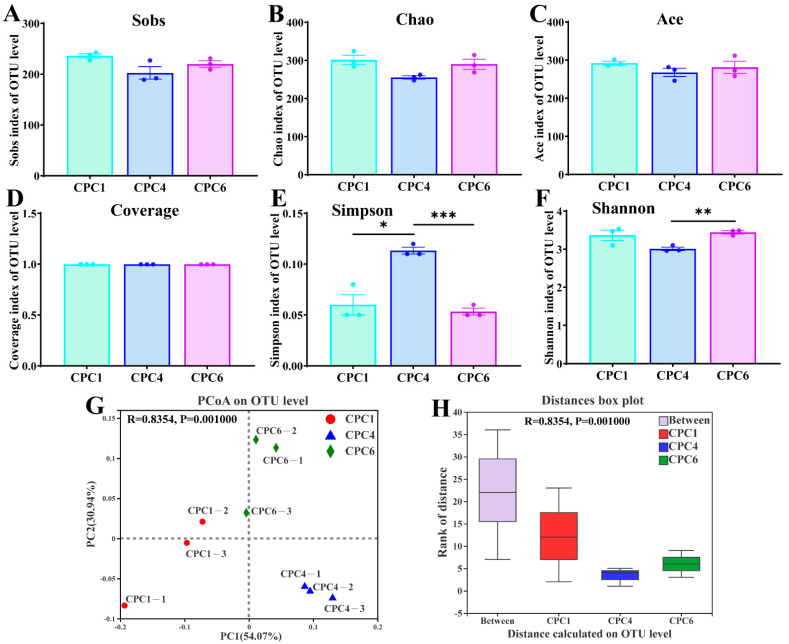
Alpha diversity analysis and Beta diversity analysis of grass carp intestinal microbiota from different dietary protein treatments. The Sobs index (**A**), Chao index (**B**), and Ace index (**C**) were used to reflect the microbial community richness. The coverage index (**D**) represents the community coverage. The Simpson index (**E**) and Shannon index (**F**) were used to reflect the microbial community diversity. Significance analysis was performed using Welch’s *t*-test, and two groups with significant differences were marked (* *p* ≤ 0.05, ** *p* ≤ 0.01, *** *p* ≤ 0.001). (**G**) Principal coordinates analysis of the weighted UniFrac scores of the microbial communities. (**H**) ANOSIM analysis (analysis of similarities) is a nonparametric test used to test whether the differences between groups are significantly greater than the differences within groups, where the “Between” box represents the between-group differences, and the other colored boxes represent the within-group differences. ANOSIM analysis showed that R = 0.8354, *p* = 0.001.

As shown in Figure 8A–C, the Sobs, Chao, and Ace indices were used to assess microbial community richness, which was not significantly affected by dietary protein levels (*p* > 0.05). In addition, alpha diversity revealed a high species coverage index (over 99%), indicating that the sequences were sufficient to capture the species richness of the samples (Figure 8D). However, microbial community diversity, including the Simpson and Shannon indices, responded to dietary protein changes (Figure 8E,F). Compared with the CPC4 group, the Simpson index was significantly lower in the CPC1 (*p* < 0.05) and CPC6 (*p* < 0.001) groups. The PCoA with weighted UniFrac distances (Figure 8G) showed that the samples were separated from each other after eight weeks of feeding with graded dietary protein levels. Further ANOSIM analysis showed that the “Between” box was higher than the boxes of the other three groups (Figure 8H), indicating that the between-group difference was more significant than the within-group difference, which proved that the grouping was statistically significant (*p* = 0.001, R = 0.8354).

**Figure 9:**
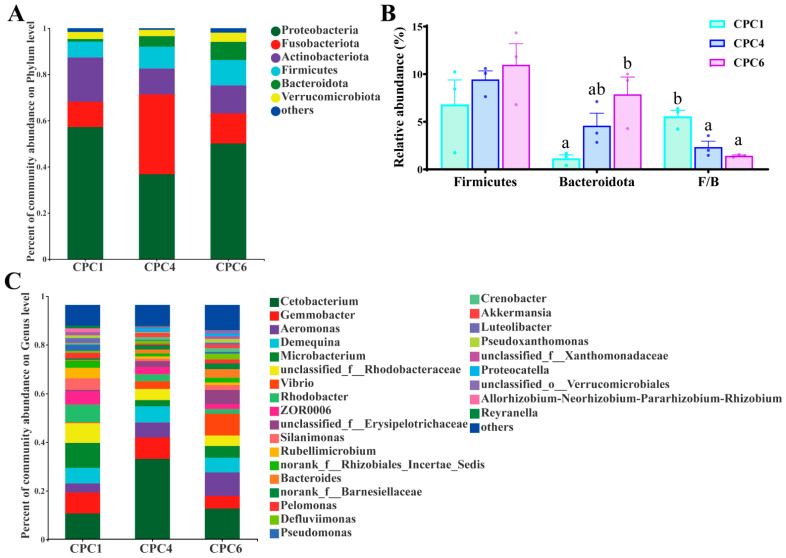
Community composition of intestinal microbiota from different dietary protein treatments. After being fed for eight weeks: the abundance of the intestinal microbial composition at the phylum (**A**) and genus (**C**) level of grass carp in the different protein level groups. Taxa with abundances <1% are included in “Others”. (**B**) One-way ANOVA significance tests were performed for Firmicutes, Bacteroides, and F/B (ratio of Firmicutes to Bacteroides). All data are the means of three parallel tanks (*n* = 3). Mean values with different superscripts were significantly different (*p* < 0.05), and the mean values without superscripts were not significantly different.

**Figure 10:**
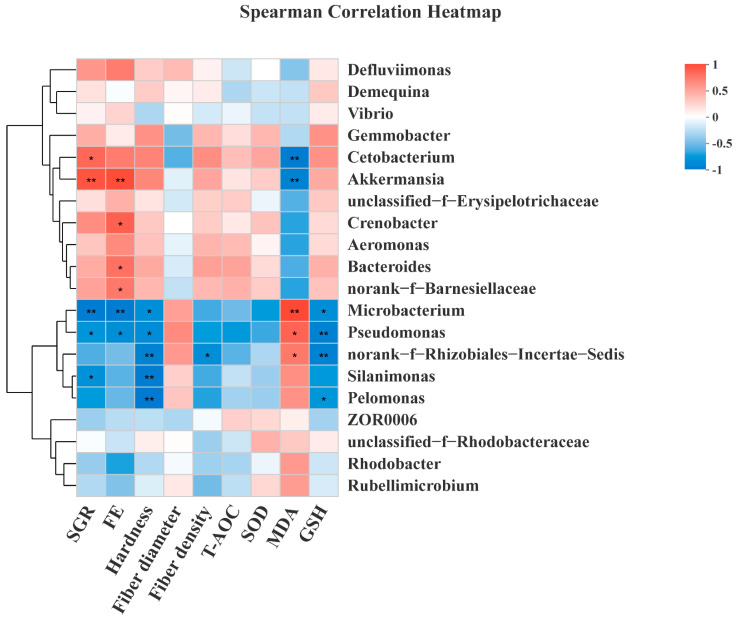
Correlation analysis of the intestinal microbiota and growth, muscle parameters. Spearman test, * *p* < 0.05, ** *p* < 0.01.

At the phylum level, this study found that the dominant phyla in the three groups were *Proteobacteria* (CPC1: 57.26%; CPC4: 36.80%; CPC6: 50.09%), *Fusobacterium* (CPC1: 10.95%; CPC4: 34.59%; CPC6: 13.07%), *Actinobacteria* (CPC1: 19.19%; CPC4: 11.20%; CPC6: 12.13%), *Firmicutes* (CPC1: 6.81%; CPC4: 9.44%; CPC6: 10.98%), and *Bacteroidetes* (CPC1: 1.15%; CPC4: 4.59%; CPC6: 7.88%) (Figure 9A). In addition, the *Bacteroidetes* showed an upward trend, and the relative abundance of the CPC6 group was significantly higher than that of the CPC1 group (*p* < 0.05). The F/B (*Firmicutes* to *Bacteroidetes* ratio) value showed a downward trend with increasing dietary protein level, and the CPC4 and CPC6 groups were significantly lower than the CPC1 group (*p* < 0.05) (Figure 9B). At the genus level, the top five genera with the highest abundance ratio were *Cetobacterium* (CPC1: 10.95%; CPC4: 34.28%; CPC6: 13.05%), *Gemmobacter* (CPC1: 8.95%; CPC4: 9.16%; CPC6: 5.37%), *Aeromonas* (CPC1: 3.89%; CPC4: 6.43%; CPC6: 10.07%), *Demequlna* (CPC1: 6.66%; CPC4: 6.88%; CPC6: 6.29%), and *Micribacterium* (CPC1: 10.67%; CPC4: 2.66%; CPC6: 5.02%) (Figure 9C). Correlation analysis (Figure 10) of the intestinal bacteria genera with grass carp growth and muscle parameters showed that SGR strongly correlates with *Cetobacterium* (r = 0.745), *Akkermansia* (r = 0.837), *Micribacterium* (r = −0.828), *Pseudomonas* (r = −0.678) and *Silanimonas* (r = −0.703). FE showed strong correlation with *Akkermansia* (r = 0.867), *Crenobacter* (r = 0.783), *Bacteroides* (r = 0.700), *norank_f__Barnesiellaceae* (r = 0.667), *Microbacterium* (r = −0.833), and *Pseudomonas* (r = −0.717). Muscle hardness was negatively correlated with *Microbacterium* (r = −0.717), *Pseudomonas* (r = −0.733), *norank_f__Rhizobiales_Incertae_Sedis* (r = −0.800), *Silanimonas* (r = −0.833), and *Pelomonas* (r = −0.850). Muscle density was also negatively correlated with *norank_f__Rhizobiales_Incertae_Sedis* (r = −0.723). Muscle MDA content was shown to correlate with *Cetobacterium* (r = −0.833), *Akkermansia* (r = −0.800), *Microbacterium* (r = 0.883), *Pseudomonas* (r = 0.767), and *norank_f__Rhizobiales_Incertae_Sedis* (r = 0.667). Muscle GSH content was shown to negatively correlate with *Microbacterium* (r = −0.717), *Pseudomonas* (r = −0.800), *norank_f__Rhizobiales_Incertae_Sedis* (r = −0.800), and *Pelomonas* (r = −0.667).

**Figure 11:**
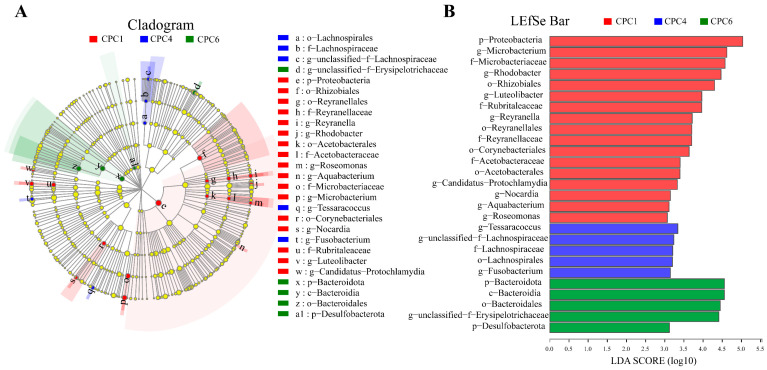
LEfSe analysis identified differential distributions of intestinal microbes among treatment groups with different dietary protein levels. (**A**) Cladogram showing the phylogenetic distribution of intestinal microbes for graded dietary protein level groups generated by LEfSe analysis (layers of the cladogram represent different levels, phyla, classes, orders, families, and genera from the inside out). (**B**) LDA scores showed bacterial differential expression between treatment groups with different dietary protein levels (LDA > 3.0, *p* < 0.05).

LEfSe analysis revealed microbial taxa enriched at different dietary protein levels (Figure 11). CPC1 (red) was mainly enriched in Proteobacteria. CPC4 (blue) was enriched in *Lachnospirales*, *Tessaracoccus,* and *Fusobacterium*. The relative abundance of Bacteroidetes dominates in CPC6 (green).

**Figure 12:**
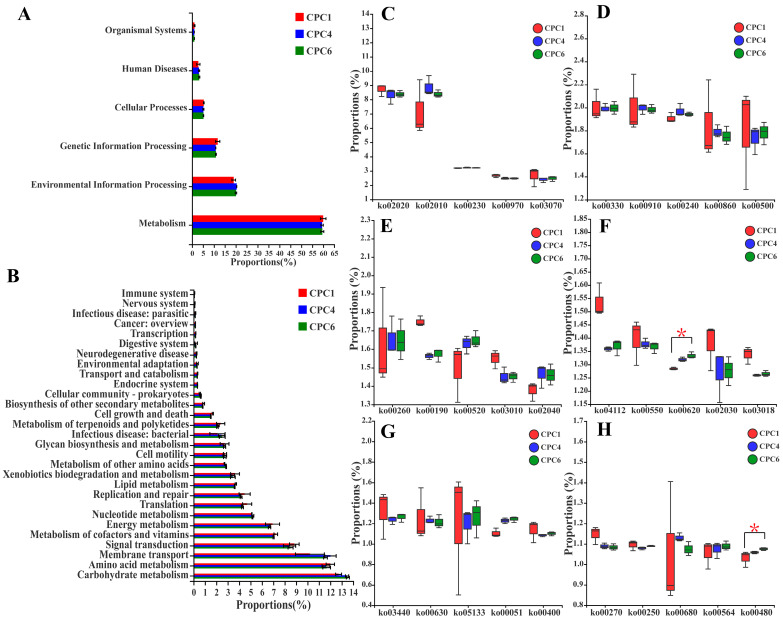
Functional prediction of the intestinal microbiota of grass carp fed with different protein level diets based on Tax4 Fun. (**A**) Functional prediction of the different treatment groups at Level 1. (**B**) Functional prediction of the different treatment groups at Level (top 30 relative abundances). (**C**–**H**) Differences in KEGG pathway abundance in the different treatment groups at Level 3 (top 30 relative abundances). Kruskal–Wallis rank-sum test was used for significance test (*n* = 3); * 0.01< *p* ≤ 0.05.

The predicted results of intestinal microbiota function are presented in Figure 12. KEGG level 1 showed that the three treatments were mainly enriched for metabolism and environmental information processing (Figure 12A). The top three functions predicted by KEGG level 2 (Figure 12B) were carbohydrate metabolism, amino acid metabolism, and membrane transport. However, significant differences were detected only between CPC1 and CPC6 in the predicted pathways of KEGG level 3 in two pathways, ko00480 (Glutathione metabolism) and ko00620 (Pyruvate metabolism) (*p* < 0.05), respectively (Figure 12C–H).

## 4. Discussion

The present study showed that the optimal dietary protein levels improved grass carp SGR and FE, while high protein levels (CPC5 and CPC6) showed relatively poor growth performance. This may be due to the depression in feeding rate and more energy expenditure on processing excess protein for deamination, increasing the nitrogen metabolism burden and affecting fish growth [28]. Based on SGR and FE, the optimal dietary protein levels for juvenile grass carp (4.68 ± 0.01 g) were estimated to be 38.61 and 38.66% in this study, respectively, which was highly consistent with the 38.63% protein requirement of grass carp (6.80 ± 0.10 g) [20]. Moreover, the result of this study was close to the 40% requirement for juvenile grass carp (4.27 ± 0.01 g) reported by Jin et al. [10], but slightly lower than the 41–43% requirement for grass carp fry (0.15–0.20 g) reported by Dabrowski [9]. This variation in dietary protein requirements is strongly associated with different life-history stages, as reported in Abdel-Tawwab et al. [27], as heavier fish reduces the protein requirements. In other words, the present study indicates that CPC is a potential material in juvenile grass carp diet, with similar nutritional value for fish growth compared with soybean meal and other traditional materials. 

In this study, PER decreased with increased protein levels, and was significantly lower in the CPC6 group compared with other groups, which was consistent with previous reports in Nile tilapia (*Oreochromis niloticus*) [27] and Songpu mirror carp [23]. Improved CF indicates good health and growth performance [10]. CF peaked in the CPC4 group in the present study, suggesting an optimal dietary protein level may promote grass carp growth by improving morphology. Previous studies have shown that HSI and MFI tend to decrease as dietary protein levels increase [10,27]. In this study, HSI and MFI first showed an increasing and then decreasing trend, which may be due to CPC use. Similar morphological parameters were reported when CPC was used to replace fishmeal in largemouth bass [4] and pearl gentian grouper [5]. The present study showed that high and low protein diets decreased dry matter digestibility, which further explains the depressed growth. In contrast, protein digestibility increased linearly, which indicates that a non-fishmeal diet (based on CPC) is tolerable for grass carp.

There was a significant negative correlation between net protein utilization (NPU) and urea levels [29]. The current study found that urea nitrogen increased linearly with the dietary protein level, suggesting that excess dietary protein can lead to protein waste. Previous studies on rainbow trout (*Oncorhynchus mykiss*) [30] and Nile tilapia [27] have shown that serum lipids tend to increase with dietary protein levels. Consistently, serum biochemical indices of triglyceride and LDL showed a trend of increasing first and then decreasing as dietary protein levels increased in this study. These serum parameters may be elevated due to converting excess protein to lipids and carbohydrates. Complement 3 and immunoglobulin M are important antibacterial compounds related to the immune response of teleost fish [11]. In this study, the content of C3 and IgM in the serum of fish showed quadratic and linear models, respectively, in response to the dietary protein level, which indicated that the CPC4 group had an improved immune ability. Consistently, the optimal protein levels could improve the immune function of the grass carp gut [11]. Similar results were also observed in mirror carp (*Cyprinus carpio*), in that the optimal protein level enhanced the C3, C4, and IgM of mirror carp at different water temperatures [31]. The immune-enhancing effect of appropriate dietary protein can be explained by increasing protein intake and feed utilization with appropriate dietary protein levels [10,27].

Oxidative damage is closely related to antioxidant enzymes and can be expressed using MDA content [32]. The current study demonstrated that optimal dietary protein levels increased the T-AOC content and GSH-Px activity while it reduced the MDA content in the hepatopancreas of grass carp, indicating that optimal dietary protein levels could protect grass carp from oxidative damage. However, the dietary protein levels did not significantly affect the hepatopancreatic SOD and CAT activities. Similarly, Xu et al. [11] reported that the optimal protein levels reduced the MDA contents but did not alter the CAT activities of grass carp in the mid intestine and distal intestine. ALT and AST are essential amino acid metabolizing enzymes and the improvement of ALT and AST reflects the vigorous activity of amino acid metabolism in fish [11,27]. In the current study, hepatopancreatic ALT and AST activities were improved in the CPC4 group, implying that amino acid metabolism was enhanced at the optimal dietary protein levels. These results suggest that the optimal protein levels for improved grass carp growth may be partly due to enhanced amino acid metabolism and improved antioxidant capacity. The hepatopancreas is an essential organ for metabolism in fish, and histological changes are considered a crucial indicator in evaluating nutritional status [19]. In the present study, fish fed CPC3 and CPC4 showed normal polygonal-shaped hepatocytes with large, clear nuclei centrally located without noticeable swelling or atrophy. These results indicate that the appropriate dietary protein level plays an essential role in promoting the antioxidant capacity and maintaining the health of the hepatopancreas. Noticeable, CPC5 and CPC6 diets depressed the hepatopancreatic ALT and AST activities and damaged the microstructure of the hepatopancreas, which indicated a reverse effect on fish metabolism and health, and possibly contributed to the depressed growth.

This study is the first to report the effect of protein levels on flesh quality when CPC was used as the protein source. Cooking loss, texture characteristics (including hardness, springiness, cohesiveness, gumminess, chewiness, resilience), and pH value are crucial parameters for evaluating muscle sensory quality [13,18]. The increasing cooking loss represents a decreased muscle water-holding capacity [12]. In this study, muscle cooking loss increased linearly with protein level, suggesting that a high-protein diet had poor muscle water-holding capacity. In addition, this study showed that the muscle pH value linearly decreased as the protein level increased. The high dietary protein group (CPC6 group) had a reduced pH value compared to the low dietary protein group (CPC1 group), which may be related to the production of more lactic acid in the muscle [12,13]. This study showed that the optimal protein level (CPC4 group) improved the texture characteristics of grass carp, such as hardness, cohesiveness, gumminess, chewiness, and resilience. Recent studies show that muscle shear force or hardness was maximized at the optimal dietary protein levels (fishmeal and casein) [12]. 

Flesh quality is often closely related to muscle fiber diameter, showing a negative correlation between the muscle fiber diameter and the hardness [14,16,19]. The larger myofiber diameters (class50, 60, and 70) accounted for more of the myofibers of fish from both the low protein level (CPC1 group) and the high protein level (CPC6 group). In contrast, the optimal protein level (CPC4 group) had smaller muscle fibers (class20, 30, and 40). Further statistical analysis showed that CPC4 had a smaller mean diameter of myofibers and a greater density of myofibers. The histological results of this study also confirmed the negative correlation between muscle fiber diameter and hardness.

In the current study, T-AOC was elevated at optimal protein levels, and the MDA content was significantly reduced, suggesting that the optimal protein levels can maintain muscle structural integrity by inhibiting oxidative damage. Further research found that the enhanced antioxidant capacity was partly attributable to the improved GSH content and SOD activity of the muscle. Differences in antioxidant capacity between treatments at different protein levels obtained in a previous study further corroborate the findings of this study [12].

After myoblasts initially form skeletal muscle, satellite cells provide additional nuclei required for skeletal muscle expansion [15]. MRFs regulate satellite cells, *myod* and *myf5* regulate satellite cell activation and proliferation, and *myog* and *mrf4* act on cell differentiation [15]. In addition, *myhc* plays a vital role in fish muscle growth by promoting myofiber proliferation and hypertrophy [16,19]. In this study, MRFs, *myhc-1,* and *myhc-4* were upregulated at the optimal dietary protein levels, indicating that appropriate dietary protein levels can effectively promote grass carp muscle growth. Moreover, optimal protein levels in this study significantly upregulated *fgf6a* and downregulated *mstn*. As reported in grass carp, the *fgf6a* plays a vital role in muscle growth regulation [16]. Besides, *mstn* has been shown to inhibit teleost muscle growth [15]. These results also confirmed that the optimal protein levels in this experiment could promote grass carp muscle growth by regulating MRFs, *fgf6a*, and *mstn*. A previous study showed that transcriptome analysis of broad bean-fed grass carp (higher hardness) muscle detected upregulation of *myog*, which plays a crucial role in promoting the formation of new muscle fibers [17]. Likewise, a recent study found that optimal dietary protein (soybean meal) levels promoted the expression of genes *myhc-1* and *myhc-4* by regulating a family of MRFs and contributed to flesh quality improvement [20]. Optimal dietary protein levels in this investigation may improve flesh quality through the same pattern. 

*Tor* regulates phosphorylation of its downstream effectors, ribosomal S6 kinase 1 (*s6k1*) and eukaryotic translation initiation factor 4 e-binding protein 1 (*4e-bp1*), which ultimately promote protein synthesis in fish and can affect *nrf2* expression as an upstream regulator of antioxidant capacity [13]. Recent studies have shown that dietary protein levels can enhance the antioxidant capacity of grass carp muscle by upregulating *tor* and *s6 k1* [12]. The optimal dietary protein level in this study may promote the muscle antioxidant capacity through the same pattern. In addition, it has been demonstrated that *igf-I* and *igf-II* promote muscle growth in hybrid catfish [15] by binding to *igf1 r*, which may indicate that dietary protein may promote muscle growth through IGFs. Still, the specific mechanism needs to be further studied. 

The intestinal microbiome has profound effects on human well-being, including host metabolism, physiology, nutrition, and immune function, and is even referred to as a “metabolic organ” [21]. Recently, studies on the regulation of intestinal microbes by dietary nutrients have received increasing attention [3,23]. In this study, the results of 16 S amplicon sequencing showed that the dietary protein decreased the intestinal microbiota diversity of grass carp, estimated using the Shannon and Simpson indices. The PCoA of the weighted UniFrac distances further revealed that CPC1, CPC4, and CPC6 were separated, and the weighted UniFrac-based ANOSIM revealed significant differences in microbiota structure between different protein levels. These findings suggest that protein levels may alter the intestinal microbiota structure of grass carp. In addition, this study identified the dominant phyla of grass carp were *Proteobacteria*, *Fusobacteriota*, *Actinobacteria*, *Firmicutes*, and *Bacteroidota*, which is consistent with the previous study on cyprinids [6,23]. Abundant *Proteobacteria* can be used to characterize intestinal microbial homeostasis, as dysregulation of homeostasis when the *Proteobacteria* abundance rises often leads to metabolic disturbances or inflammation [21]. A previous study of Songpu mirror carp reported that the abundance of *Proteobacteria* increases with protein levels [23]. This is partly consistent with the result between the CPC4 and CPC6 groups. The abundance of *Proteobacteria* was reduced at the optimal protein level (CPC4), which may benefit the homeostasis of intestinal microbiota.

More and more attention has been paid to studying the abundance ratio (F/B ratio) of *Firmicutes* and *Bacteroides* [33]. In humans and mice, increased F/B ratios are often associated with obesity, diabetes, and metabolic disorders [33]. In this study, the F/B value of the CPC4 group decreased significantly and showed good growth performance and health status, which provided a reference for studying the relationship between F/B and health. A decrease in the F/B ratio with increasing protein levels was consistently observed in Songpu mirror carp [23]. *Firmicutes* can extract energy from food [33]. Increased dietary protein levels in the present study promoted the abundance of *Firmicutes*, suggesting that the better weight gain in CPC4 and CPC6 groups than the low-protein group (CPC1 group) may be attributed to increased energy generation and utilization. The LEfSe analysis showed that the abundance of *Bacteroides* increased in response to the protein levels and contributed significantly to the differences in the CPC6 group. It is reported that *Bacteroidetes* have carbohydrate-related enzymes, and *Bacteroidetes* and *Firmicutes* play an essential role in the energy metabolism and glucose metabolism of organisms [34]. Combined with the apparent digestibility data, these results may imply that more energy is available for growth in the CPC4 group than in the CPC1 group. Still, the increase in ADC_p_ in the CPC6 group and the decrease in ADC_d_ indicate that less energy is available for growth in the CPC6 group than the CPC4 group.

At the genus level, *Cetobacterium* was the most abundant in the gut of grass carp through community composition maps, consistent with previous studies on freshwater fish [3,23]. *Cetobacterium* is reported to produce vitamin B12 and can ferment peptides and carbohydrates, and inhibit the growth of harmful bacteria [35]. *Akkermansia* represents a novel biomarker of intestinal metabolic health coupling and is essential for treating metabolic syndrome [36]. Spearman’s correlation analysis showed that *Cetobacterium* and *Akkermansia* were strongly associated with growth performance while negatively correlated with muscle MDA content. These results well demonstrate the excellent growth performance of CPC4 in this study and explain its possible assistance to the antioxidant capacity of muscle. In contrast, *Microbacterium* and *Pseudomonas* exhibited a negative correlation with SGR, muscle hardness, and muscle GSH content while they were strongly positively correlated with muscle MDA content. These results confirmed that *Microbacterium* is one of the most closely related cornerstone genera of other bacteria and plays a crucial role in the growth and health of fish [37], and *Pseudomonas* is one of the most important opportunistic pathogens of grass carp [6]. In this study, in addition to being associated with growth and health, these genera also affected muscle hardness and antioxidant capacity. The interrelationship between intestinal microbiota and growth, health, and flesh quality requires more research to elucidate its specific regulatory mechanisms.

Intestinal microbiota affects the host mainly through its metabolites. The phylum *Fusobacterium* can metabolize carbohydrates to butyrate [34,38], which mediates the regulation of intestinal inflammatory processes, atherosclerosis, and immune system maturation [39]. In addition, among *Firmicutes*, the *Lachnospiraceae*, *Lactobacillaceae*, and *Ruminococcaceae* species hydrolyze starch and other sugars to produce butyrate and other short-chain fatty acids (SCFAs) [39], which inhibit the growth of harmful bacteria. In this study, *Fusobacterium* and *Lachnospiraceae* were significantly enriched in the CPC4 group, suggesting that optimal protein levels may further contribute to the health of grass carp by enhancing the abundance of SCFA-producing bacteria. However, the mechanism of their specific interactions needs further study.

The functional prediction of grass carp intestinal microbial communities based on Tax4 Fun revealed that, interestingly, at KEGG pathway level 3, ko00480 (Glutathione metabolism) and ko00620 (Pyruvate metabolism) showed significant differences between the CPC1 and CPC6 groups. Glutathione metabolism is part of amino acid metabolism and contains important antioxidant molecules to protect the body from oxidants [22]. A previous study showed that grass carp fed low protein levels had a lower intestinal GSH-Px and GSH content than the high protein levels [11]. Similarly, ko00480 (Glutathione metabolism) was significantly downregulated in the low protein level treatment group in this study, suggesting that a diet below the optimum protein requirement may impair amino acid metabolism functions and the antioxidant system. Cells convert glucose to pyruvate in the cytoplasmic matrix through glycolysis. Pyruvate can produce a large amount of adenosine triphosphate (ATP) under aerobic conditions. In contrast, pyruvate can produce lactate and a small amount of ATP through anaerobic glycolysis under anoxic conditions [40]. The significant enrichment of the ko00620 (Pyruvate metabolism) pathway in the CPC6 group may indicate that grass carp respond to a high-protein diet by enhancing glucose metabolism to obtain more energy to meet the needs of metabolizing protein. However, the mechanism of action between animal intestinal microbiota and metabolic function requires more studies.

Microcrystalline cellulose is the most commonly used filler and binder in fish feed [41]. Previous studies on the dietary protein requirements of red drum (*Sciaenops ocellatus*) [42] and dietary carbohydrate-to-lipid ratios of channel catfish (*Ictalurus punctatus*) [43] reached 15.56 and 40.61% microcrystalline cellulose use, respectively, which suggest that the use of a varying amount of cellulose in protein requirement studies is acceptable. In addition, a recent study also showed that using 1.84–31.84% microcrystalline cellulose in grass carp diets did not produce adverse effects [41]. This study was conducted to meet the demand for extruded feed in the aquatic feed market by producing extruded feed, and varying starch levels may disable the production of extruded diets at high starch levels, so microcrystalline cellulose was used as a filler to make the extruded feed. This might result in a relatively high optimal protein level. However, when comparing the optimal protein level with other results in grass carp, this study is acceptable.

## 5. Conclusions

In short, the optimal dietary protein level for juvenile grass carp provided by CPC was estimated to be 38.61–38.66%. Optimal dietary CPC levels promote grass carp performance, health, and flesh quality. In addition, optimal dietary CPC levels help maintain intestinal microbiota homeostasis. The results of this study demonstrate that CPC is a protein source with potentially significant application given its similar nutritional value compared to traditional protein sources. Altogether, the results of this study provide a theoretical basis for the development of sustainable and high-performance aquafeeds for valuable aquaculture species. However, based on the growth results in this study, we should pay attention to the amino acid composition of the CPC raw materials and fish carcasses, and explore the amino acid balance technology to better the CPC utilization in the future. In addition, the specific components of CPC that can play a role in affecting intestinal flora, and their specific mechanisms of regulating intestinal flora growth and health, are also worth exploring.

## Figures and Tables

**Table 1:** Ingredients and proximate composition of the experimental diets with different protein levels (expressed as % dry matter).

	CPC1	CPC2	CPC3	CPC4	CPC5	CPC6
Cottonseed protein concentrate (CPC) ^1^	38.40	44.50	50.70	56.90	63.10	69.2
Soy oil:Fish oil (1:1)	5.90	5.76	5.62	5.47	5.32	5.14
Corn starch	18.00	16.80	15.60	14.40	13.20	11.98
Microcrystalline cellulose	34.30	29.54	24.68	19.83	14.98	10.29
Vitamin and mineral premix ^2^	1.00	1.00	1.00	1.00	1.00	1.00
Calcium biphosphate	1.80	1.80	1.80	1.80	1.80	1.80
Choline chloride (50%)	0.50	0.50	0.50	0.50	0.50	0.50
Yttrium oxide	0.10	0.10	0.10	0.10	0.10	0.10
Proximate composition (%)						
Moisture	6.55	7.14	6.67	7.42	7.42	7.88
Crude protein (dry matter)	24.80	30.51	33.68	37.69	41.43	45.61
Crude lipid (dry matter)	7.92	8.04	7.91	7.95	7.97	8.02
Ash (dry matter)	5.70	6.52	6.67	7.15	7.63	8.29

^1^ The CPC was provided by Xinjiang Jinlan Plant Protein Co., Ltd., Shihezi, Xinjiang, China, which contained 61.51% crude protein, 2.36% crude lipid, 5.35% moisture, 5.70% ash, 4.82% crude fiber, 19.55 MJ/kg gross energy, and 285 mg/kg of free gossypol. The essential amino acid contents in the CPC were (g/kg) Lysine, 22.6; Methionine, 9.7; Arginine, 68.6; Histidine, 16.4; Leucine, 30.6; Isoleucine, 16.4; Valine, 23; Tryptophan, 8.1; Phenylalanine, 29.8; and Threonine, 15.9. ^2^ Each kilogram of the vitamin and mineral premix contained: L-ascorbate-2-monophosphate (35%), 900 mg; vitamin E, 450 mg; inositol, 225 mg; nicotinamide, 120 mg; calcium pantothenate, 60 mg; vitamin A, 30 mg; vitamin K3, 30 mg; vitamin B2, 22.5 mg; vitamin B6, 22.5 mg; vitamin D3, 15 mg; vitamin B1, 15 mg; folic acid, 15 mg; vitamin B12, 120μg; biotin, 3 mg; ferrous sulfate monohydrate, 300 mg; zinc sulfate/sulphate monohydrate, 200 mg; Sodium chloride, 100 mg; manganese sulphate, 25 mg; copper (II) sulfate pentahydrate, 30 mg; cobaltous chloride (10% Co), 5 mg; sodium selenite (10% Se), 5 mg; potassium iodate (2.9%), 3 mg; and magnesium sulphate, 900 mg.

**Table 2:** Real-time PCR primer sequences.

Genes ^1^	Forward Primer (5′-3′)	Reverse Primer (5′-3′)	Amplification Efficiency (%)	Accession Number
*myod*	ATGGAGTTGTCGGATATTCCCTTC	GCGGTCAGCGTTGGTTGTT	104.45	MG544985
*myog*	TTACGAAGGCGGCGATAACTT	TGGTGAGGAGACATGGACAGA	101.18	JQ793897
*myf5*	GTGCCTGTGCCTCATCTCCT	AATGCGTGGTTCACCTTCTTCA	92.41	GU290227
*mrf4*	TCGCTCCTGTATTGATGTTGATGA	GCTCCTGTCTCGCATTCGTT	107.98	KT899334
*fgf6a*	CGCATACGAGTCTTCCAT	CCTACGAGAACATCCAACA	102.95	MK050993
*fgf6b*	TCCAGTCCGCTTCCGAGTA	AGATGAAACCCGATGCCTACA	91.14	MK050992
*mstn*	CTGACGCCAAGTTCCACATACA	CGACTCTGCTTCAAGTTCTTCTCT	99.15	KP719016
*myhc-7*	AACTGCGCTGTAACGGTGTA	AGTGTGCCCAAACCTGTACT	101.85	MW113233
*myhc-2*	ACAGTGGCCAGCATTGATGA	TCCGCAGAGTTCAAACCCAA	101.15	MW113235
*myhc-4*	ACTCCGCTGACATGCTGAAA	TGTCCAGCACACCAATGAAGA	103.78	MW113236
*myhc-1*	TTCCGTTGTTGTGTCAGGCT	TACTGGATGACGCGTTTGGT	99.12	MW113234
*igf-II*	TCTGTGGCAGTCCTCAACAAC	TTCCGCAACTTCTTCGCTCTT	97.78	EF062860
*tor*	TCCCACTTTCCACCAACT	ACACCTCCACCTTCTCCA	105.68	JX854449
*s6k1*	ACATAAAGCAGCCTGACG	TGGAGGAGGTAATGGACG	101.51	EF373673
*4e-bp1*	GCTGGCTGAGTTTGTGGTTG	CGAGTCGTGCTAAAAAGGGTC	99.59	KT757305
*β-actin*	TATGTTGGTGACGAGGCTCA	GCAGCTCGTTGTAGAAGGTG	98.82	M25013
*ef1α*	TGACTGTGCCGTGCTGAT	CGCTGACTTCCTTGGTGATT	99.51	GQ266394

^1^*myod*—myogenic differentiation antigen; *myog*—myogenin; *myf5*—myogenic factor 5; *mrf4*—muscle regulatory factor 4; *fgf6a*—fibroblast growth factor 6 a; *fgf6b*—fibroblast growth factor 6 b; *mstn*—myostatin; *myhc-7*—myosin heavy chain 7; *myhc-2*—myosin heavy chain 2; *myhc-1*—myosin heavy chain 1; *myhc-4*—myosin heavy chain 4; *igf-II*—insulin-like growth factor 2; *tor*—target of rapamycin; *s6k1*—ribosomal protein S6 kinase 1; *4e-bp1*—4 e-binding protein 1; *ef1α*—elongation factor 1-alpha.

**Table 3:** Effects of dietary protein levels on the growth, apparent digestibility, and morphological parameters of grass carp ^1^.

Items ^2^	Diet Treatments						PSE ^3^	Orthogonal Contrast ^4^			Regression		
	CPC1	CPC2	CPC3	CPC4	CPC5	CPC6		Linear	Quadratic	Cubic	Model ^5^	(Pr > F) ^6^	R^2^
IBW (g)	4.70	4.69	4.70	4.67	4.67	4.67	0.03	0.903	0.447	0.281	Ns	-	-
FBW (g)	11.83 ^a^	12.39 ^ab^	14.72 ^bc^	15.42 ^c^	14.61 ^bc^	13.11 ^abc^	1.23	0.040	0.004	0.234	Qd	0.003	0.541
SR (%)	96.67	97.78	97.78	95.56	97.78	97.78	3.77	0.858	0.870	0.630	Ns	-	-
SGR (%/d)	1.66 ^a^	1.72 ^a^	2.04 ^b^	2.13 ^b^	2.03 ^b^	1.84 ^ab^	0.16	0.030	0.005	0.216	2 SBL-LL	0.003	0.887
FR (%/d)	4.09 ^b^	3.50 ^a^	3.63 ^a^	3.55 ^a^	3.44 ^a^	3.44 ^a^	0.18	0.001	0.060	0.087	Ln	0.004	0.410
FE (%)	38.58 ^a^	46.81 ^ab^	51.72 ^b^	54.78 ^b^	54.26 ^b^	50.18 ^b^	5.03	0.005	0.009	0.872	2 SBL-LL	0.000	0.982
PER	1.56 ^b^	1.53 ^b^	1.54 ^b^	1.45 ^b^	1.31 ^ab^	1.10 ^a^	0.15	0.001	0.078	0.750	Ln	0.000	0.528
ADC_d_ (%)	66.89 ^c^	72.02 ^d^	71.18 ^d^	71.60 ^d^	61.51 ^b^	56.27 ^a^	1.85	0.000	0.000	0.214	Qd	0.000	0.881
ADC_p_ (%)	64.43 ^a^	67.03 ^a^	69.86 ^ab^	70.62 ^ab^	75.99 ^bc^	79.77 ^c^	3.54	0.000	0.409	0.698	Ln	0.000	0.734
CF (%)	1.70 ^a^	1.77 ^ab^	1.84 ^bc^	1.89 ^c^	1.84 ^bc^	1.79 ^abc^	0.05	0.032	0.003	0.680	Qd	0.001	0.603
HSI (%)	1.86 ^a^	2.14 ^cd^	2.22 ^d^	2.28 ^d^	2.02 ^bc^	1.91 ^ab^	0.08	0.917	0.000	0.187	Qd	0.000	0.777
MFI (%)	1.29 ^a^	1.52 ^b^	1.85 ^c^	2.25 ^d^	2.40 ^e^	1.58 ^b^	0.04	0.000	0.000	0.000	Qd	0.000	0.741

^1^ All data are the means of three parallel tanks (*n* = 3). Mean values not sharing a common superscript in the same row are significantly different (*p* < 0.05), while mean values in the same row without any superscript are not different. Growth parameters, feed utilization (except PER), and digestibility parameters were calculated with reference to the study of Wang et al. [20], PER was calculated according to the study of Abdel-Tawwab et al. [27] and morphological parameters were calculated according to the study of Abouel Azm et al. [16]. ^2^ IBW, initial body weight; FBW, final body weight. SR (Survival rate, %) = 100 × (number of survival/total number). SGR (Specific growth rate, %/d) = 100 × (ln (FBW)–ln (IBW))/(experimental period (d)). FE (Feed efficiency, %) = 100 × [FBW (g)–IBW (g)]/dry feed intake (g). FR (Feeding rate, %/d) = 100 × dry feed intake/(experimental days × (FBW + IBW)/2). PER (Protein efficiency ratio) = (FBW (g) – IBW (g))/(dry feed intake (g) × dietary protein content). ADC_d_ (Apparent digestibility coefficient of dry matter, %) = 100 × (1 – (dietary yttrium content/fecal yttrium content)). ADC_p_ (Apparent digestibility coefficient of protein, %) = 100 × (1 – (dietary yttrium content/fecal yttrium content) × (fecal protein content/dietary protein content)). HSI (Hepatosomatic index, %) = 100 × (final hepatopancreas weight (g)/final body weight (g)). MFI (Mesenteric fat index, %) = 100 × (mesenteric fat weight (g)/final body weight (g)). CF (Condition factor, %) = fish weight (g) × 100/fish length (cm)^3^. ^3^ PSE = Pooled standard error of treatment means (*n* = 3). ^4^ If statistical significance (*p* < 0.05) was detected, the model that fits best with the data was selected. ^5^ Ns = No structure (*p* > 0.05); 2 SBL-LL = Two slope broken line-linear ascending and linear descending; Ln = Linear; Qd = Quadratic. ^6^ Probability associated with the F-statistic test.

**Table 4:** Effects of dietary protein levels on the body and dorsal muscle proximate composition of grass carp (% fresh weight) ^1^.

	Diet Treatments						PSE ^2^	Orthogonal Contrast ^3^			Regression		
	CPC1	CPC2	CPC3	CPC4	CPC5	CPC6		Linear	Quadratic	Cubic	Model ^4^	(Pr > F) ^5^	R^2^
Whole body													
Moisture	72.03	72.54	72.38	71.55	72.38	71.32	0.70	0.180	0.317	0.869	Ns	-	-
Crude Protein	13.65	13.68	13.77	13.77	13.72	13.64	0.24	0.951	0.415	0.863	Ns	-	-
Crude Lipid	9.90	9.95	10.84	11.12	10.85	10.80	0.45	0.005	0.051	0.416	Ln	0.007	0.375
Ash	3.14	3.33	2.66	3.13	2.59	3.09	0.41	0.344	0.360	0.357	Ns	-	-
Dorsal muscle													
Moisture	77.20	76.68	76.19	77.03	77.45	77.13	0.72	0.434	0.249	0.130	Ns	-	-
Crude Protein	18.18	18.69	18.68	18.72	18.35	18.23	0.34	0.649	0.026	0.368	Qd	0.049	0.331
Crude Lipid	3.72	3.89	3.88	3.88	3.77	3.63	0.31	0.610	0.252	0.876	Ns	-	-
Ash	0.44	0.45	0.46	0.39	0.44	0.50	0.05	0.514	0.197	0.181	Ns	-	-

^1^ All data are the means of three parallel tanks (*n* = 3). Mean values not sharing a common superscript in the same row are significantly different (*p* < 0.05), while mean values in the same row without any superscript are not different. ^2^ PSE = Pooled standard error of treatment means (*n* = 3). ^3^ If statistical significance (*p* < 0.05) was detected, the model that fits best with the data was selected. ^4^ Ns = No structure (*p* > 0.05); Ln = Linear; Qd = Quadratic. ^5^ Probability associated with the F-statistic test.

**Table 5:** Effects of dietary protein levels on biochemical indices and immune parameters in the serum of grass carp ^1^.

	Diet Treatments						PSE ^3^	Orthogonal Contrast ^4^			Regression		
Indices ^2^	CPC1	CPC2	CPC3	CPC4	CPC5	CPC6		Linear	Quadratic	Cubic	Model ^5^	(Pr > F) ^6^	R^2^
TP (g/L)	38.00	38.57	38.17	38.63	40.97	42.00	2.43	0.036	0.322	0.945	Ln	0.021	0.291
GLU (mmol/L)	5.60	5.00	4.70	4.97	5.43	5.67	1.10	0.726	0.236	0.665	Ns	-	-
UN (mmol/L)	0.50 ^a^	0.53 ^a^	0.63 ^a^	0.77 ^ab^	1.07 ^bc^	1.10 ^c^	0.17	0.000	0.391	0.355	Ln	0.000	0.696
TG (mmol/L)	2.50 ^a^	2.85 ^a^	3.57 ^b^	3.99 ^b^	3.68 ^b^	3.57 ^b^	0.35	0.000	0.004	0.448	Qd	0.000	0.710
CHOL (mmol/L)	6.70	6.95	7.17	6.75	6.67	6.60	0.63	0.567	0.424	0.530	Ns	-	-
HDL (mmol/L)	2.69	2.70	2.73	2.75	2.78	2.77	0.32	0.202	0.778	0.776	Ns	-	-
LDL (mmol/L)	4.22 ^a^	4.37 ^ab^	4.61 ^b^	4.44 ^ab^	4.33 ^ab^	4.30 ^ab^	0.18	0.884	0.027	0.334	Qd	0.069	0.300
C3 (μg/mL)	122.62 ^a^	125.61 ^ab^	127.58 ^ab^	130.02 ^bc^	133.96 ^c^	124.87 ^ab^	2.87	0.016	0.005	0.025	Qd	0.008	0.471
IgM (μg/mL)	32.20 ^a^	32.29 ^a^	38.00 ^b^	38.83 ^b^	38.42 ^b^	40.42 ^b^	2.08	0.000	0.199	0.751	Ln	0.000	0.650
LYS (ng/mL)	181.00	187.22	178.87	179.95	180.99	176.60	6.50	0.230	0.660	0.737	Ns	-	-

^1^ All data are the means of three parallel tanks (*n* = 3). Mean values not sharing a common superscript in the same row are significantly different (*p* < 0.05), while mean values in the same row without any superscript are not different. ^2^ TP, total protein; GLU, glucose; UN, urea nitrogen; TG, triglyceride; CHOL, cholesterol; HDL, high-density lipoprotein; LDL, low-density lipoprotein; C3, complement 3; IgM, immunoglobulin M; LYS, lysozyme. ^3^ PSE = Pooled standard error of treatment means (*n* = 3). ^4^ If statistical significance (*p* < 0.05) was detected, the model that fits best with the data was selected. ^5^ Ns = No structure (*p* > 0.05); Ln = Linear; Qd = Quadratic. ^6^ Probability associated with the F-statistic test.

**Table 6:** Effects of the dietary protein levels on the muscle texture of grass carp ^1^.

	Diet Treatments						PSE ^2^	Orthogonal Contrast ^3^			Regression		
	CPC1	CPC2	CPC3	CPC4	CPC5	CPC6		Linear	Quadratic	Cubic	Model ^4^	(Pr > F) ^5^	R^2^
Cooking loss (%)	29.38 ^a^	31.17 ^ab^	33.78 ^abc^	35.54 ^bc^	35.58 ^bc^	38.16 ^c^	2.71	0.001	0.677	0.781	Ln	0.000	0.611
Hardness (g)	1037.24 ^a^	1188.03 ^b^	1353.18 ^c^	1476.93 ^d^	1151.90 ^ab^	1104.84 ^ab^	65.95	0.289	0.000	0.854	Qd	0.000	0.687
Springiness	0.53	0.51	0.48	0.48	0.52	0.50	0.10	0.448	0.145	0.442	Ns	-	-
Cohesiveness	0.46 ^a^	0.47 ^ab^	0.48 ^abc^	0.49 ^bc^	0.50 ^c^	0.47 ^ab^	0.00	0.020	0.013	0.106	Qd	0.007	0.480
Gumminess	504.58 ^a^	557.33 ^ab^	660.12 ^bc^	741.68 ^c^	683.29 ^c^	557.53 ^ab^	59.94	0.028	0.000	0.065	Qd	0.000	0.637
Chewiness (g)	279.51 ^a^	325.41 ^b^	367.64 ^c^	433.60 ^d^	313.62 ^b^	305.25 ^ab^	15.19	0.051	0.000	0.663	Qd	0.000	0.645
Resilience (g/s)	0.26 ^c^	0.24 ^bc^	0.22 ^ab^	0.21 ^a^	0.22 ^ab^	0.23 ^ab^	0.00	0.004	0.001	0.794	Qd	0.000	0.717
pH	5.40 ^b^	5.34 ^ab^	5.31 ^ab^	5.30 ^ab^	5.31 ^ab^	5.22 ^a^	0.09	0.042	0.918	0.361	Ln	0.023	0.282

^1^ All data are the means of three parallel tanks (*n* = 3). Mean values not sharing a common superscript in the same row are significantly different (*p* < 0.05), while mean values in the same row without any superscript are not different. ^2^ PSE = Pooled standard error of treatment means (*n* = 3). ^3^ If statistical significance (*p* < 0.05) was detected, the model that fits best with the data was selected. ^4^ Ns = No structure (*p* > 0.05); Ln = Linear; Qd = Quadratic. ^5^ Probability associated with the F-statistic test.

## Data Availability

Data is contained within the article.
